# Association between telomere length and idiopathic normal pressure hydrocephalus: a Mendelian randomization study

**DOI:** 10.1002/alz.088201

**Published:** 2025-01-03

**Authors:** Feng Yang, Hanlin Cai, Meng Yi Ren, Keru Huang, Hui Gao, Linyuan Qin, Ruihan Wang, Ping Yong Chen, Liangxue Zhou, Qin Chen

**Affiliations:** ^1^ West China Hospital of Sichuan University, Chengdu China; ^2^ west china hospital, chengdu, sichuan China

## Abstract

**Background:**

Idiopathic normal pressure hydrocephalus (iNPH) is highly prevalent among elderly individuals, and there is a strong correlation between telomere length and biological aging. However, there is limited evidence to elucidate the relationship between telomere length and iNPH. This study aimed to investigate the associations between telomere length and iNPH using the Mendelian randomization (MR) method.

**Method:**

The genetic variants of telomere length were obtained from 472,174 UK Biobank individuals. Summary level data of iNPH were acquired from 218,365 individuals of the FinnGen consortium. Five MR estimation methods, including inverse‐variance weighting (IVW), MR‐Egger regression, weighted median, weighted mode and simple mode, were used for causal inference. Comprehensive sensitivity analyses were conducted to test the robustness of our results. In addition, multivariable MR was further implemented to identify potential mechanisms in the causal pathway from telomere length to iNPH.

**Result:**

Genetically determined longer telomere length was significantly associated with decreased risk of iNPH (OR = 0.44, 95% CI 0.24‐0.80; P = 0.008). No evident heterogeneity (Cochran Q = 138.11, P = 0.386) and pleiotropy (MR Egger intercept = 0.01, P = 0.514) were observed in the sensitivity analysis. In addition, multivariable MR indicated that the observed association was attenuated after adjustment for several vascular risk factors, including essential hypertension (IVW OR = 0.55, 95% CI 0.30‐1.03; P = 0.061), type 2 diabetes (IVW OR = 0.71, 95% CI 0.09‐5.39; P = 0.740) and coronary artery disease (IVW OR = 0.58, 95% CI 0.31‐1.07; P = 0.082).

**Conclusion:**

Our MR study revealed a strong negative correlation of telomere length with iNPH. The causal relationship might be driven by several vascular risk factors.

